# Complete genome sequence of *Cedecea davisae* 739Q*,* a newly isolated strain from patient’s ulcer secretion

**DOI:** 10.1128/mra.01334-25

**Published:** 2026-01-15

**Authors:** Wei Lu, Lihong Li, Yunuo Wu, Xinxin Zhou, Daojun Yu, Shenghai Wu

**Affiliations:** 1 Department of Laboratory, Affiliated Hangzhou First People's Hospital, School of Medicine, Westlake University 557712, Hangzhou, China; 2 The Fourth School of Medicine Affiliated to Zhejiang Chinese Medical University, Hangzhou, China; Rochester Institute of Technology, Rochester, New York, USA

**Keywords:** *Cedecea davisae*, opportunistic pathogens

## Abstract

We report the complete genome of *Cedecea davisae* 739Q obtained from the ulcer secretions of a clinical patient. The genome has one circular chromosome (4.95 Mb) and one circular plasmid (76.9 Kb). These sequencing data provide a solid groundwork for future studies.

## ANNOUNCEMENT

*Cedecea davisae* is an uncommon opportunistic pathogen belonging to the family Enterobacteriaceae. It is described as an emerging pathogen, which can cause bacteremia in patients (1–3). Human clinical specimens are usually obtained from the respiratory tract. However, the clinical significance is unknown. In addition, *Cedecea davisae* has the ability to degrade various organic matter and is resistant to colistin and cephalosporin (4). To better understand the functions of this important microbial virulence and resistance as well as its ability to promote pathogenicity at the genomic level, we report the complete genome sequence of *Cedecea davisae* 739Q.

The 739Q isolate was isolated from the discharge of an ulcer in a patient with diabetic foot in September 2024 and identified by matrix-assisted laser desorption/ionization time of flight mass spectrometry (MALDI-TOF MS) in microbiological testing laboratory (5). Isolate 739Q was incubated at 37°C overnight, and genomic DNA was extracted using the DNeasy PowerLyzer Microbial Kit (Qiagen Co., MI, USA) based on the manufacturer’s protocol. Next, 300 ng extracted DNA was used for subsequent sequencing. The Illumina sequencing library was prepared using the EpiNext DNA Library Preparation Kit, and sequencing was performed on the Illumina MiSeq platform (2 × 300 bp, 500 cycles). A total of 3,278,365 reads and 967.7 Mbp of sequencing data were generated. Nanopore sequencing was conducted for 96 h on the MinION platform using flow cell R9.4.1, producing 44,516 reads with an N50 read length of 13,962 bp, totaling to 289.7 Mbp. Quality control of the raw sequencing data from the Illumina and Nanopore platforms was conducted using fastp 0.23.4 (6) and NanoPlot 1.43.0 (7), respectively. The long-read sequencing data were used for *de novo* genome assembly with Flye 2.9.5 (8), resulting in a single circular contig with an average coverage of 47×. The contig was then polished using Medaka 2.0.1 (Oxford Nanopore Technologies, Ltd.) and Pilon 1.24 (9) with Illumina short reads.

**Fig 1:**
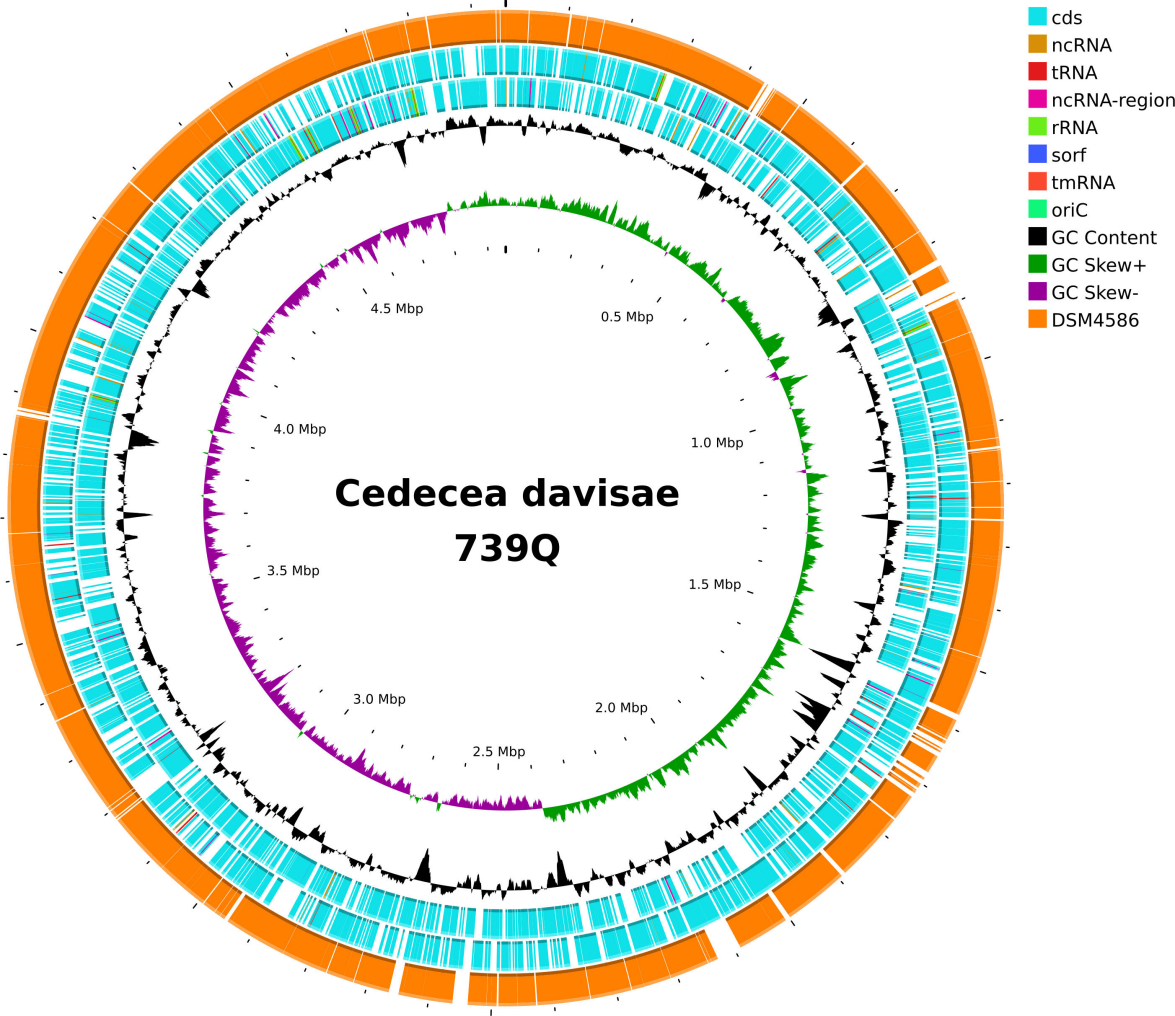
Chromosome features of *Cedecea davisae* 739Q. From outside to inside: circle 1, representation of the *Cedecea davisae* DSM4586 genome; circles 2 and 3, predicted ORFs encoded in the plus and minus strands, respectively; circles 4 and 5, GC content and GC skew maps, respectively; and circle 6, scale in kb (each tick is 0.1Mb).

The genome assembly had a completeness of 100% and a contamination level of 0.01%, as confirmed by CheckM version 1.2.3 (10). The assembled genome consists of a single contig of chromosome 4,954,274 bp with a G+C content of 54.35% and a single contig of plasmid 76,940 bp with a G+C content of 53.96% (Table 1 and Fig. 1). The genome was annotated using the NCBI Prokaryotic Genome Annotation Pipeline (11). 739Q contained 4,691 genes, including 4,586 protein-coding genes, 22 rRNA genes, 82 tRNA genes, and one ncRNA. ResFinder 4.6.0 determined that antimicrobial resistance genes were absent in the 739Q genome (12). Strain 739Q shared over 98.61% average nucleotide identity with the reference strain DSM 4568 (accession number: GCA_000412335.2) and was confirmed as *Cedecea davisae* by FastANI (13). This study for the first time assembled the complete genome sequence of *Cedecea davisae* (Fig. 1).

**TABLE 1:** Sequencing and assembly data of *Cedecea davisae* 739Q strain

Parameter	Chromosome	Plasmid
Isolated location	China	
Time	24 September 2024	
Type	Skin ulcer	
Assembly size (bp)	4,954,274	76,940
Gap number	0	0
Number of contigs	1	1
Topology	Circular	Circular
GC content (%)	54.35	53.96
Genome coverage	1.00	6.32
Number of 5S rRNA	12	0
Number of 16S rRNA	3	0
Number of 23S rRNA	3	0
Number of tRNAs	24	0
Total number of CDS	4,567	102
Completeness (%)	100	100
Contamination (%)	0.001	0.001
GenBank accession no.	CP172054.1	CP172053.1

## Data Availability

This complete genome sequence has been deposited in GenBank under accession numbers CP172054.1 and CP172053.1. Raw sequencing data were deposited in the NCBI Sequence Read Archive (SRA) database under BioProject no. PRJNA1201644 and BioSample no. SAMN45940028. The SRA accession numbers are SRX27179316 (ONT) and SRX27179315 (Illumina).
